# Adolescents’ perception of gender differences in bullying

**DOI:** 10.1111/sjop.12523

**Published:** 2019-01-28

**Authors:** Lisa Hellström, Linda Beckman

**Affiliations:** ^1^ Department of School Development and Leadership Malmö University Malmö Sweden; ^2^ Department of Public Health Karlstad University Karlstad Sweden

**Keywords:** Adolescents, bullying, focus groups, gender

## Abstract

Gender norms are normative societal expectations regarding the behaviors of girls and boys that can guide bullying behavior. As early adolescence is a time when peer relations become increasingly important, it is critical to understand the peer relationships of adolescents and what is considered gender non‐confirming behavior. Therefore, the aim of this study is to analyze Swedish girls’ and boys’ perception of gender differences in bullying. Twenty‐one Swedish adolescents (8 girls and 13 boys) took part in four focus group discussions separated by boys and girls. Data analysis was conducted using qualitative content analysis. “Expectations and needs to fit the norm” emerged as the main category as all categories emerging from the analysis related to boys’ and girls’ understandings of how expectations, strategies, expressions relating to bullying and the need to belong vary depending on gender. Further, girls and boys expressed admiration for each other's ways of coping with bullying indicating that also coping strategies are associated with expectations based on gender. For schools and adults to be better equipped to meet the needs of girls and boys and understand how these needs are expressed, adolescents voices regarding gender related bullying can be seen as helpful tools to develop strategies to work with gender norms and gender expectations. In light of the results of our study, schools may have work to do when it comes to the awareness of norms and attitudes and how they are expressed as these may be a foundation for bullying, among both staff and students.

## Introduction

What is considered acceptable behavior among adult women and men is deeply rooted in expectations surrounding girls and boys from a young age. Gender norms can be defined as social expectations of appropriate behaviors for men as compared to women (Pulerwitz & Barker, 2008). Often, these gender norms guide actions and are values and attitudes formed in society, in school, on the internet, in sports clubs or any other environment including young girls and boys. Societal gender norms restrict individuals to gender identities and expressions that conform to the male‐female binary (Oswald, Blume & Marks, 2005). Research on the role of gender in bullying indicates that girls and boys who show higher levels of gender non‐conformity are at higher risk of bullying during adolescence (e.g., Aspenlieder, Buchanan, McDougall & Sippola, 2009; D'Augelli, Grossman & Starks, 2006). Children and adults do not always share the same understanding of bullying meaning that adults may not always intervene when children experience bullying. Hence, students’ perception regarding bullying could be the critical missing component in the undertaking of understanding and addressing bullying in schools (Gamliel, Hoover, Daughtry & Imbra, 2003; Varjas, Meyers, Bellmoff, Lopp, Birckbichler & Marchall, 2008). As early adolescence is a time when peer relations become increasingly important, it is critical to understand the peer relationships of adolescents and what is considered gender non‐confirming behavior (Toomey, Card & Casper, 2014). Therefore, the aim of this study is to analyze Swedish adolescents’ perception of gender differences in bullying.

### Previous research on gender differences in bullying

Gender patterns in bullying and aggressive expressions have been evident over time. Bullying, defined as repetitive aggressive behaviors with an intent to be harmful or hurtful including some form of power imbalance between those involved (Olweus, 1996) can take the form of direct verbal or physical aggression, relational aggression such as rumor spreading or gossiping, either online or offline (Wang, Iannotti & Nansel, 2009). Early studies and a recent meta‐analysis (Casper & Card, 2017) concluded that boys were more overtly victimized (direct, physical) than girls showing a small to medium effect size, while there was no significant difference between boys and girls regarding relational victimization. In a meta‐analysis by Cook, Williams, Guerra, Kim and Sadek (2010), the correlations of gender and the roles of bully, victim and bully/victim were reported. While the analysis showed a stronger correlation for boys to be bullies compared to girls (small to medium effect size), the role of gender concerning victim rates was found less consistent and the differences between boys and girls were small, but still showed that boys were more commonly reported as a victim compared to girls (very small effect size). When it comes to bully‐victims, Cook *et al*. (2010) found a stronger correlation for boys compared to girls, but the effect size was still considered small.

Research has tried to explain gender differences in bullying by stating that boys are more socialized to use direct physical aggression, whereas girls have learned to use less aggression, or to vent their aggression indirectly (Björkqvist, 1994; Lagerspetz, Björkqvist & Peltonen, 1988). Girls seem to engage in fewer and closer friendships, while boys report friendships that are more casual and greater in number (Lagerspetz *et al*., 1988; Maccoby, 2003). As a result of this structure, girls may choose indirect (relational) aggression over overt as it targets these relations and are seen as particularly hurtful (Galen & Underwood, 1997; Underwood, Galen & Paquette, 2001). Girls’ way of spreading rumors, gossip and excluding other girls could be a strategy to achieve close friendships with other girls and reduce the risk of exclusion (Espelage, Meban & Swearer, 2004). Further, masculinity has been shown to predict bullying behavior (Gini & Pozzoli, 2006; Navarro, Larrañaga & Yubero, 2011). This link might be explained by cultural representations, values, and social expectations (Gini & Pozzoli, 2006). It has been suggested that bullying is a powerful process of social control and that the perpetrator seeks power and leadership within the peer group (Olweus, 1993; Smith & Brain, 2000). As shown in previous studies (Phoenix, Frosh & Pattman, 2003), it seems that for many boys masculinity is important to achieve in order to avoid being bullied. As children grow up and develop their cognitive ability they can learn gender stereotypical values from observing acts of different men and women models. Girls who endorse aggressive behavior may stand in too big contrast to the normative view of girl behavior to be accepted. While physical aggression among boys is more acceptable, and even expected if you want to be popular (Espelage *et al*., 2004; Guerra, Kirk, Williams & Sadek, 2011), physically aggressive girls, on the other hand, may be perceived as immature and norm breaking (Eliasson, 2007). Gender differences in aggression may vary depending on normative expectations and the anticipated consequences of aggression, that is, if boys display non‐normative behavior, they may suffer social consequences because of it (Bem, 1995; Card, Stucky, Sawalani & Little, 2008). Similarly, numerous researches have shown that coping strategies among boys and girls differ and that they have different experiences and concerns regarding risks. Accordingly, boys and girls tend to have different views about seeking adult help (Newman, 2008). Girls tend to seek social support more often compared to boys who regularly show reluctance towards using peer and adult support and talking to someone if bullied (Boulton, Boulton, Down, Sanders & Craddock, 2017; Cowie, 2000; Hunter, Boyle & Warden, 2004; Naylor, Cowie & del Rey, 2001).

According to the Swedish Educational Act (SFS, 2010:800) boys and girls have equal rights and possibilities to develop and all have the right to feel safe in school. Still, conscious and unconscious gender norms may inhibit these rights and can serve as a basis for bullying, harassment and discrimination (e.g., Smith & Leaper, 2006). Despite schools’ responsibility and ambitious efforts to prevent bullying and promote gender equality, expectations of behavior based on gender still exist, both from adolescents themselves as well as from adults. As students’ perception regarding bullying could be the critical missing component in the undertaking of understanding and addressing bullying in schools (Gamliel *et al*., 2003; Varjas *et al*., 2008), the aim of this study is to analyze Swedish adolescents’ perception of gender differences in bullying.

## Methods

### Data and participants

This study is part of a larger mental health‐promoting project among school children (“The Preventive School project”) and is based on data collected in the spring of 2012 in Karlstad, Sweden. The projects’ objective was to study health related factors in an adolescent population, with a focus on bullying. The study involves students in the ages 13 and 15 (Grades 7 and 9) from two different schools. Twenty‐one students (8 girls and 13 boys) took part in focus group discussions. A total of four focus group discussions were performed compiling four girls from Grade 7 (13 years of age), four girls from Grade 9 (15 years of age), seven boys from Grade 7 (13 years of age) and six boys from Grade 9 (15 years of age) into separate groups. The ethics committee at Karlstad University, Sweden has reviewed the project without any objections (reference number C2012/193).

### Procedure

Two schools were selected to be included in the study after agreement from the responsible principals. The schools were chosen because their large size was expected to provide a great variety and selection of students. The students and parents were given written information in advance and the students were informed that their participation was voluntary, that their answers were anonymous, and that they could terminate their participation at any point. The parents of the students in Grade 7 were asked to sign a written consent for their child's participation in the study. For students in Grade 9, parental consent was not required. All students in Grade 7 and 9 at the two schools were asked to contact their class teacher if they wanted to participate in a focus group discussion. All students who wanted to participate were invited to a focus group discussion. The discussions were held at the school of the students and were about bullying in general and the students were not actively encouraged to talk about gender differences. The main questions of interest in the focus group discussions were “What does bullying mean to you?” and “What do you think is the reason that people are bullied?” The moderator asked the questions and followed up with questions such as “can you develop what you just said,” “what do you mean” and “can you give any examples.” Before the focus groups ended the students were asked if they had anything to add or if they thought that something important had been left out of the discussion.

### Analysis

Data analysis was conducted using qualitative content analysis (Graneheim & Lundman, 2004). Each focus group was transcribed verbatim and quotations were sorted to find patterns in the statements of the adolescents. The transcription of each focus group was read through numerous times by both authors and meaning‐carrying units responding to the aim of the study were extracted. Descriptions of gender differences constituted the unit of analysis. In the next step, the meaning‐carrying units were condensed and abstracted into codes. In order to identify similarities and differences the codes were compared and then sorted into subcategories. As the analysis proceeded, subcategories were subsequently clarified and adjusted and one main category emerged. The initial coding of the transcripts was performed by the first author, and the coded data were examined by the second author for emergent subcategories. The interpretations were compared and discussed until consensus was reached. Comparisons were made with the context in each step of the analysis, to verify the empirical base of the data.

## Results

### Expectations and needs to fit the norm

Analysis of the focus group discussions resulted in one main category and three subcategories. “Expectations and needs to fit the norm” emerged as the main category as all categories emerging from the analysis related to boys’ and girls’ understandings of how expectations, strategies, expressions relating to bullying and the need to belong vary depending on gender. Subcategories included “bullying to achieve power,” “coping with bullying” and “behavior expectations based on gender.”

### Bullying to achieve power

According to the boys in the study, you seldom see a single girl bully another girl. Bullying among girls often includes smaller groups bullying each other or someone within the group:

I think that girls often attack in groups. Instead of … you seldom see a girl, one‐to‐one so to speak (boy, age 13).

Just as the boys, the girls perceive themselves to socialize in smaller groups and that the urge to belong can make you do bad things such as talk bad about someone or give mean gazes:

Among girls in fourth class, I guess … fourth or fifth class, then you want to be with this one person really bad, and then you can be really mean to someone else (girl, age 13).

Further, the girls expressed that bullying could be used as a tool to achieve power:

For girls it does not always have to be the weak ones. Because it could be the strongest in the group who attacks someone else who is strong. Just because … They do it to seem cool. I mean, that is how it goes. It becomes … everything is about group pressure (girl, age 15).

It emerged that boys perceive themselves to be more straightforward and physical compared to girls. Boys perceive themselves to use violence and to alleviate their aggression physically instead of going behind each other's back and talk bad about someone. The aggression could be a result of boys’ temper:

It doesn't have to take long for boys. It could just be a couple of words … then you … [snaps with his fingers] you snap. You get mad as hell (boy, age 15).

According to the girls in the study, boys hang out in larger groups and are more straightforward and physical compared to girls. Boys in the same class form a group where everyone can be with each other. According to the girls, boys who are different stand out and may not be accepted into the class community:

I really think that boys go after the weak guy, the one … the small guy … the immature guy (girl, age 15).

According to the girls, you seldom see other girls bully boys, especially not boys in a group:

It is easier to oppress a girl, that's why girls attack other girls (girl, age 13).

However, being a target of boys’ bullying behavior was also perceived to increase the risk of a girl to be bullied by other girls.

### Coping with bullying

For boys as well as girls, the group works as an important support system when coping with bullying. Girls are perceived to be better at talking to their friends if they have been bullied which the boys interpret as it being easier for girls to not take it as seriously and that girls do not have as much to lose in a fight:

The girls still have a lot left after a fight with someone. Boys have a lot more to lose, I think (boy, age 15).

On the other hand, boys also see girls as being more resentful and stubborn compared to themselves:

Eventually, it could be that the girls ignore this girl completely and she becomes bullied behind her back (boy, age 13).

Compared to boys, girls consider themselves to be fussier, more excluding and not as confrontational. The girls discuss that their less confrontational approach could be that they are not as honest and straight forward compared to boys or that it would be a much bigger deal:

If a girl would shout … shout right out, then it would be interpreted much differently. It would be like a mean thing right away (girl, age 13).

It was also expressed that girls may be more sensitive and hold on to mean comments longer making them feel worse as a result.

The boys further mention that all boys have their pride and should not let anyone tell them what to do and that they often trigger and excite each other within the group. Backing down is not an alternative:

When you do take the fight, there is no turning back. You can't back down because then you become weaker (boy, age 15).

That means that boys may avoid conflicts to a greater extent because they know that it could lead to physical violence. The girls in the study described that boys can say what they think without it being taken too seriously. They are more tolerant compared to girls:

I think that girls make a bigger deal out of it. If someone says that you have an ugly hat, then a girl would walk around thinking about it … //if you don't have a friend or thinking that they don't like you then perhaps more people don't like you … but boys would probably not care about it and would just say it straight out (girl, age 13).

Girls mention that even though boys may be perceived to not be upset when being bullied, they think that ignoring mean comments or dismissing them as playing around may just be a way of handling the situation:

I think that many girls make a bigger deal about it. Boys probably have an easier time just ignoring it (girl, age 13).

Further, boys are considered not to be afraid of conflicts:

Boys are not as afraid of conflicts, I think. Girls can say something and walk away while boys are more like … they stay and start a fight if necessary (girl, age 15).

### Behavior expectations based on gender

According to the boys, girls who like activities typically seen as masculine stand out in a negative way, for example, girls who like to play ice hockey. The prejudice is that girls are expected to like fashion and wear makeup. The girls described that the expectations on them did not only concern being more restrained and not getting into fights but also to be nice, calm, proper, neat and to care about their looks.

Boys’ perception is that they have to win every fight because that is built in the expectations of being male – you have to be the best and you cannot back down even an inch. Because of the expectations, it is easier to detect boys who are weak. It emerged that another expectation is that boys should like to do sports and masculine things:

If a boy is very aware of fashion, perhaps likes to read magazines and to wear certain clothes … then, if you stick out and dares to try different things … much is labeled what is what. I mean, if it's a boy's thing or a girl's thing. It's the same thing in society. And if you dare to cross over to the other side, maybe try something different then you become hesitant because then everyone will know. That you are doing something … like horseback riding for a boy for example. Then everyone will be at his throat (boy, age 15).

According to the girls, boys who are good at different sports are positively perceived. Boys are expected to be physically and mentally strong and it is also more accepted if they fight and are loud:

If two boys fight, it is forgotten the next day (girl, age 15).

If boys would bully a girl, it would be because she would not act in a way that a girl is supposed to:

Boys, if they would bully a girl it would be because she is cocky and acts in a boyish way. Because … I mean, my own experience. I was bullied by boys. Because I was … I was like a boy. Because … I would rather go up to someone and say things straight up than say it behind their back. So to speak. And I couldn't … if they started to harass me then I could walk up to them and say “what do you want?” So it really depends on the type of girl’ (girl, age 15).

## Discussion

Our main findings showed that the boys and girls in our study have a very stereotypical perception of gender differences in bullying and that different expectations and needs to fit the norm are perceived to explain these gender differences. Based on the statements of the adolescents, it is evident that gender plays an important role in the expectations on how to behave and what strategies are used in coping with bullying. The literature confirms most of our results. “Bullying to achieve power” was exemplified by girls’ desire to form stronger relationship and reach higher status by excluding other girls, which is seen in earlier research (Espelage *et al*., 2004; Galen & Underwood, 1997; Underwood *et al*., 2001). This indicates that the context might have a more powerful effect for girls due to their heightened concern about social consequences for their behavior (Boldizar, Perry & Perry, 1989). In that way, social exclusion can be seen as a powerful tool for girls to get what they want. For boys, their own perception was that they use bullying as a tool to alleviate aggression and that the easiest target for this aggression were the “weak guys.” These results are in line with previous research showing that boys way of displaying aggression depend on normative expectations and the anticipated consequences of aggression, that is, if boys display non‐normative behavior, they may be seen as easier targets (Bem, 1995; Card *et al*., 2008). Both boys and girls in the current study seem to use bullying as a tool to feel a sense of belonging, to prevent being excluded from the group for the girls and prevent being perceived as weak for the boys.

In “Coping with bullying,” girls and boys saw different characteristics among each other that they interpreted as beneficial when dealing with bullying. While girls were seen by boys as better at talking to their friends, girls perceived boys’ ways of ignoring incidences or mean comments as being able to handle more. Girls considered boys not to avoid conflicts, while the boys, on the contrary, expressed conflict avoidance because it could lead to physical violence. Hence, avoidance expressed by the boys in the current study may be used as a strategy to minimize the risk of victimization. This is confirmed in recent research showing that feelings of rejection put boys at an increased risk of being victimized (Didaskalou, Roussi‐Vergou & Andreou, 2017). What is interesting is that boys and girls seem to admire each other's different strategies for coping with bullying. Avoidance strategies as the ones expressed by the boys in the current study have proven to be least successful and more likely to increase the victimization over time (Didaskalou *et al*., 2017). A meta‐analysis on coping strategies concludes that coping skills programs aimed at components that minimize stress in the broader environment or that promote social support are more successful when it comes to psychosocial functioning for youth compared to strategies that place more responsibility on the individual (Clarke, 2006). The results from the current study indicate that adolescents may need help to develop alternative coping strategies with the help of adults.

In the category “Behavior expectations based on gender” the young people in our study believed that girls who like to do typically masculine activities were more likely to be victimized, which is also confirmed elsewhere (Bacchini, Affuso & Trotta, 2008). Similarly, our participants felt that boys bully other boys who are weak, and thus do not show their masculinity as expected in a masculine culture (Connell, 1995). Similar to other studies stating that socialization processes and norms embedded in the school context perpetuate different normative and socially accepted behavior for boys and girls (Bussey & Bandura, 1999; Chesney‐Lind, 1989), we found girls to have expectations on them including not getting into fights but also to be nice, calm, proper, neat and to care about their looks. In contrast, both boys and girls believed that boys are expected to like sports and masculine activities and to be physically and mentally strong. These expectations can be seen as demands in that sense that they were expressed as a burden to the teenagers.

### Applied implications

Despite schools’ hard work, educational efforts among staff, and laws and regulations concerning bullying, these types of behaviors still exist. If bullying is assumed to be rooted in gender norms, then the failure to eradicate bullying may be because schools are not tackling such norms. Adolescents’ expression of gender norms could indicate shortcomings and can be seen as one way to guide schools in their work to promote peer relations and gender equality. Peer relations are important for the display of prosocial behavior among adolescents (Oldfield, Humphrey & Hebron, 2016) and a large peer group may work as a protection from being selected as targets of bullying (Wang *et al*., 2009). According to the adolescents in the current study, the need to belong is expressed somewhat differently depending on gender and could partly explain bullying behavior. For schools and adults to be better equipped to meet the needs of girls and boys and understand how these needs are expressed, adolescents’ voices regarding gender related bullying can be seen as helpful tools to develop strategies to work with gender norms and gender expectations. Schools and adults’ strategic and systematic work with bullying may be based on an understanding of what it means to be young from an adult's perspective that is not in line with the understanding or the expressed needs of children and youth (Frisén, Holmqvist & Oscarsson, 2008; Hopkins, Taylor, Bowen & Wood, 2013; Maunder, Harrop & Tattersall, 2010). Previous research has concluded that the risk of not including the understanding of children and youth is that many incidences with hurtful or harmful consequences may go unnoticed by adults (Hellström, Persson & Hagquist, 2015). This study adds to this conclusion suggesting that adolescents’ understandings can be used in the preventive anti‐bullying work.

Further, girls and boys in the current study expressed admiration for each other's ways of coping with bullying indicating that also coping strategies are associated with expectations based on gender. Adolescents may need help with developing alternative tools to handle expectations as well as needs to fit the norm. In the current study, boys expressed coping strategies that are based on the expectations to be seen as strong and how to avoid being seen as weak and for the girls coping were expressed as a question of how to form stronger friendships. These survival strategies may stem from girls’ and boys’ different friendship structures where girls socialize in smaller groups and boys in larger groups (Lagerspetz *et al*., 1988; Maccoby, 2003). The girls and boys in the current study expressed strength with these structures, for example, having someone to talk to (girls) and having someone to back you up (boys). However, these structures may also trigger certain behaviors, such as social exclusion for girls, and no room for individual feelings or showing strength in other ways than physical aggression for boys. How adults approach the task of “toughening up” boys may limit the potential for creating safe schools as it perpetuates the existing gender norms (Serriere, 2008). One way for adults to guide adolescents in developing alternative tools might be to create opportunities for girls to interact in larger groups and for boys to interact in smaller groups, to provide alternative ways of belonging. Role models and the opportunity to engage in larger groups may encourage girls to speak their minds and to form alternative friendships. Girls, as well as boys, need to be included in the analysis and planning of activities that promote positive peer relations (SNAE, 2011), but need the guidance from adults in terms of a clear framework and structure.

### Methodological considerations and future studies

This study uses a small sample of adolescents and the results are not directly generalizable to other countries or age groups. The current study used focus groups to encourage active discussions. The group interaction offered by focus groups encourage people to talk to one another; asking questions, exchanging experiences and commenting on each other's points of view (Kitzinger, 1994). As perceptions of gender differences were in focus in the study, the division of boys and girls into separate groups made the group a safe place to discuss bullying and girls’ and boys’ perception of each other (Horowitz, Vessey, Carlson, Bradley, Montoya & McCullough, 2003). Choosing girls and boys from different schools and grade levels also enhanced the credibility of the data as it offered a richer variation and understanding of how the phenomenon of bullying is expressed among girls and boys in various ages and in different contexts. Further, the trustworthiness was enhanced by involving two researchers in the analysis process to reach consensus and by including quotations from the transcribed text showing similarities within and differences between categories (Graneheim & Lundman, 2004). The peer dynamics and relations between the participants in the focus groups were not known. Previous negative relations between group members could have had an impact on the content of discussions. Adolescents’ perceptions on gender differences in bullying were identified but the participants were not asked about their personal experiences with bullying. Hence, we do not know whether the participants had been bullied or had bullied others which could have affected their perceptions of bullying. When group members have no personal experience with the topic, their discussions are based on opinions, which questions the transferability of the results in the current study to other groups and contexts (Graneheim & Lundman, 2004; Horner, 2000). This study was conducted among adolescents in the ages 13–15 years. To use children's understandings of gender differences in bullying as a tool for schools’ anti‐bullying work starting in the early school years, it would be interesting to conduct a similar study among children in younger ages. In addition, similar studies among adults could give insights concerning adults and school personnel's understanding of gender expectations and gender norms and how the anti‐bullying work could be conducted on a more structural level.

### Conclusions

Our results show that gender stereotypical perceptions among adolescent girls and boys are present in their every‐day life. These perceptions may stem from the need to belong and need to fit the norm, which is expressed differently among girls and boys. The way girls and boys cope with bullying may also stem from expectations based on gender indicating that adolescents may need help with developing alternative tools to handle expectations as well as needs to fit the norm. In light of the results of our study, schools may have work to do when it comes to the awareness of norms and attitudes and how they are expressed, among both staff and students.
